# Progressive muscle proteome changes in a clinically relevant pig model of Duchenne muscular dystrophy

**DOI:** 10.1038/srep33362

**Published:** 2016-09-16

**Authors:** Thomas Fröhlich, Elisabeth Kemter, Florian Flenkenthaler, Nikolai Klymiuk, Kathrin A. Otte, Andreas Blutke, Sabine Krause, Maggie C. Walter, Rüdiger Wanke, Eckhard Wolf, Georg J. Arnold

**Affiliations:** 1 Laboratory for Functional Genome Analysis (LAFUGA), Gene Center, LMU Munich, Feodor-Lynen-Str. 25, D-81377 Munich, Germany; 2 Chair for Molecular Animal Breeding and Biotechnology, Gene Center and Department of Veterinary Sciences, LMU Munich, Feodor-Lynen-Str. 25, D-81377 Munich, Germany; 3 Institute of Veterinary Pathology, Centre for Clinical Veterinary Medicine, LMU Munich, Veterinärstr. 13, D-80539 Munich, Germany; 4 Friedrich-Baur-Institute, Department of Neurology, LMU Munich, Ziemssenstr. 1, D-80336 Munich, Germany

## Abstract

Duchenne muscular dystrophy (DMD) is caused by genetic deficiency of dystrophin and characterized by massive structural and functional changes of skeletal muscle tissue, leading to terminal muscle failure. We recently generated a novel genetically engineered pig model reflecting pathological hallmarks of human DMD better than the widely used *mdx* mouse. To get insight into the hierarchy of molecular derangements during DMD progression, we performed a proteome analysis of biceps femoris muscle samples from 2-day-old and 3-month-old DMD and wild-type (WT) pigs. The extent of proteome changes in DMD vs. WT muscle increased markedly with age, reflecting progression of the pathological changes. In 3-month-old DMD muscle, proteins related to muscle repair such as vimentin, nestin, desmin and tenascin C were found to be increased, whereas a large number of respiratory chain proteins were decreased in abundance in DMD muscle, indicating serious disturbances in aerobic energy production and a reduction of functional muscle tissue. The combination of proteome data for fiber type specific myosin heavy chain proteins and immunohistochemistry showed preferential degeneration of fast-twitch fiber types in DMD muscle. The stage-specific proteome changes detected in this large animal model of clinically severe muscular dystrophy provide novel molecular readouts for future treatment trials.

Duchenne muscular dystrophy (DMD; OMIM reference 310200) affects 1 in 3,600–6,000 live male births and is caused by mutations (mainly large genomic deletions) in the X-linked dystrophin gene (*DMD*). The absence of the essential muscle protein dystrophin (DMD) results in progressive muscle degeneration and wasting. Most patients are diagnosed around 5 years of age and are wheelchair-bound before their teens. Without intervention, the mean age at death is around 19 years (reviewed in ref. 1). Though no curative treatment for DMD is currently available, several promising genetic approaches are under development (reviewed in ref. 2).

Different animal models have been used to dissect disease mechanisms of DMD and to test therapeutic strategies (reviewed in refs 3, 4, 5). The most widely used animal model of DMD is the *mdx* mouse which has a nonsense mutation in exon 23 of the *Dmd* gene. In addition, several other strains with different *Dmd* mutations, including a targeted deletion of exon 52^6^, have been developed (reviewed in ref. 7). Recent studies demonstrated the feasibility of exon skipping^8^ and gene-editing approaches in the *mdx* mouse^9^,^10^,^11^.

Skeletal muscle samples of *mdx* mice have been extensively studied by holistic profiling at the transcriptome^12^,^13^,^14^,^15^,^16^ and proteome levels^17^,^18^,^19^,^20^,^21^,^22^,^23^,^24^,^25^,^26^,^27^ to unravel molecular derangements caused by dystrophin deficiency and to assess the consequences of different treatment strategies at a molecular level. Early proteome studies of *mdx* hindlimb muscles used two-dimensional gel electrophoresis and mass spectrometry to identify differentially abundant proteins compared to wild-type (WT) mice^27^ and to investigate the time course of proteome changes at different ages^17^. Another 2D-gel based analysis addressing calcium-binding proteins in the muscle proteome of *mdx* mice^25^, further enlightened a disturbed calcium handling in DMD. A fluorescence difference in-gel electrophoretic (2D-DIGE) study of gastrocnemius muscle samples from 6-week-old *mdx* vs. WT mice found reduced levels of proteins involved in glycolysis, whereas proteins involved in the citric acid cycle and electron transport chain as well as structural proteins were increased in abundance in *mdx* vs. WT samples^26^. Subsequent proteome studies addressed age-related changes in the *mdx* tibialis anterior muscle^24^, differences between various muscles^23^, and age-related changes in the diaphragm, the muscle most severely affected in the *mdx* mouse^28^. In contrast, extraocular muscles of *mdx* mice are spared from major pathology, and a 2D-DIGE analysis revealed only moderate proteome changes compared to the corresponding WT muscles^29^. This was confirmed by the first gel-free proteome study of spared extraocular muscles vs. affected diaphragm from 2-month-old *mdx* mice and age-matched WT controls^30^. A recent study using stable isotope labeling in mouse (SILAC mouse) compared gastrocnemius muscle samples of 3-week-old *mdx* and WT mice and quantified 789 proteins, of which 73 were significantly different in abundance between the two genotypes^21^. Ontology analysis of the differently abundant proteins suggested the integrin-linked kinase pathway, actin cytoskeleton signaling, mitochondrial energy metabolism, and calcium homeostasis to be involved in the early pathology related to dystrophin deficiency. A multi-omics approach combining proteomics, mRNA and microRNA data of tibialis anterior tissue of WT, untreated *mdx* mice and *mdx* mice treated by exon skipping^22^ detected 525 differentially abundant proteins related to various pathways, amongst them some related to mitochondrial energy production, TCA cycle, amino acid degradation, gluconeogenesis and fatty acid metabolism. Another more recent study, comparing proteomes of mildly vs. severely affected *mdx* mouse muscle^20^, found a fibrosis-related increase of collagen and a decrease in calcium binding proteins in severely affected muscles. Furthermore, annexins, lamins and vimentin were categorized as universal dystrophic markers. A drawback of the *mdx* mouse model is that it shows– except for the diaphragm – no severe muscle pathology and has a near normal life span (reviewed in ref. 31). Therefore various double-knockout mice were generated, from which e.g. utrophin/dystrophin deficient mice^32^ display several clinical features of human DMD.

A further frequently used animal model is the Golden Retriever muscular dystrophy (GRMD) model^33^ which shows a much more severe muscle pathology than the *mdx* mouse and better reflects the clinical course of human DMD. A recent proteomics study of vastus lateralis muscle samples from 4-month-old GRMD dogs and healthy controls by isotope-coded affinity tag (ICAT) profiling revealed that mainly proteins involved in metabolic pathways were decreased in abundance in GRMD muscle^34^, suggesting defective energy metabolism as a hallmark of the disease. GRMD cranial sartorius muscle displayed increased levels of myotrophin and spectrin as compared to age-matched normal dogs, providing a possible explanation for the relative hypertrophy and cytoskeletal stability of this particular muscle in GRMD^35^.

A drawback of this model is that the phenotype is highly variable (reviewed in refs 5 and 36), possibly caused by epigenetic effects and modifier genes^37^.

We recently generated a tailored pig model of DMD, which is deficient of *DMD* exon 52 and thus resembles a frequent mutation in human DMD^38^. DMD pigs lack dystrophin in skeletal muscles and show clinical signs of a severe myopathy, including elevated serum creatine kinase levels, progressively impaired mobility and muscle weakness, and a maximum life expectancy of 3 months due to respiratory failure. While heart muscle samples of DMD pigs did not show any severe pathology, histological examination of skeletal muscles and diaphragm revealed excessive fiber size variation, numerous large rounded hypertrophic fibers, branching fibers and fibers with central nuclei, as well as segmentally necrotic fibers, hypercontracted fibers and groups of small regenerating muscle fibers. These lesions were associated with interstitial fibrosis and mononuclear inflammatory cell infiltration, mimicking the hallmarks of the human disease. The severity and extent of these alterations progressed with age^38^. Morphometric analyses of biceps femoris muscle samples revealed that the mean minimal Feret’s diameter of muscle fibers was 34% and 55% reduced in 2-day-old and 3-month-old DMD pigs as compared to age-matched wild-type (WT) pigs. Furthermore, the proportion of muscle fiber profiles with centrally located nuclear profiles was doubled in 2-day-old DMD pigs and was increased by more than 20-fold in 3-month-old DMD pigs compared to age-matched WT controls, indicating a rapidly progressing DMD pathology^38^.

The present study used this unique and well-characterized set of samples to identify progressive proteome changes in skeletal muscle of a large animal model of DMD, reflecting genetic, biochemical, histological and clinical hallmarks of the human disease and revealing molecular readouts for the efficacy of future treatment trials.

## Results

### Overview of identified proteome changes

**Figure 1:**
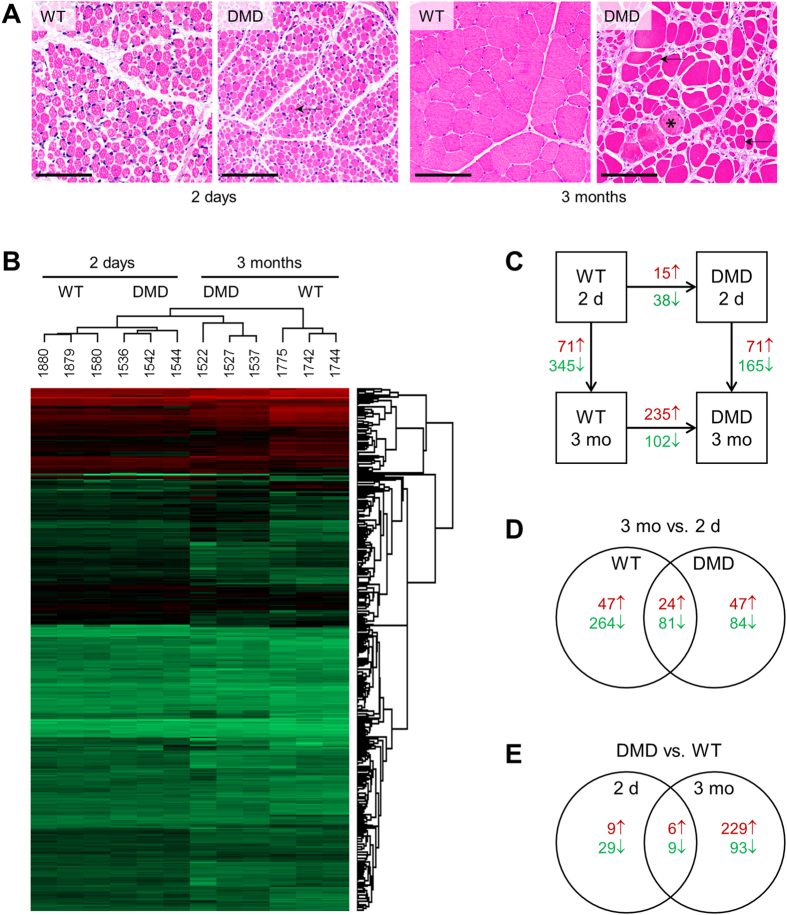
Progressive pathology and associated proteome changes in biceps femoris muscle of DMD pigs. (**A**) Histology of biceps femoris muscle in WT versus DMD pigs at 2 days and 3 months of age. Note the variation in fiber diameter, rounded fibers, fibers with internalized central nuclei (arrows), and muscle fiber necrosis (*) in DMD pigs. Between 2 days and 3 months of age, there is a pronounced increase in the diameter of muscle fibers in both groups, indicating muscle growth by fiber hypertrophy. Plastic (GMA/MMA) sections, hematoxylin and eosin (H&E) staining; bars = 100 μm. (**B**) Hierarchical clustering of label free quantitation (LFQ) values clearly separates four groups according to age (2 days vs. 3 months) and genotype (DMD vs. WT). (**C**) Numbers of differentially abundant proteins between genotypes within age (horizontal arrows) and between ages within genotype (vertical arrows). Differences were considered to be significant at an adjusted p-value < 0.05 and a log2-fold change > |0.6|. Numbers of proteins with significantly increased (red) and decreased (green) abundance between the group at an arrowhead and the group at the end of the respective arrow are shown. (**D**) Numbers of significant age-related protein abundance differences in WT and DMD pigs. Note that there is only a small overlap of age-related proteome changes between the two genotypes. (**E**) Numbers of significant protein abundance differences between DMD and WT pigs at the age of 2 days and 3 months. Note that there is only a small overlap of proteome changes induced by dystrophin deficiency in the two age classes.

We performed liquid chromatography tandem mass spectrometry (LC-MS/MS) approach combined with a label free quantification (LFQ) analysis of biceps femoris muscle samples from 2-day-old and 3-month-old DMD and WT pigs. Representative histological sections of the investigated muscle samples from the four groups are shown in Fig. 1A. A progressive muscular dystrophy in DMD pigs and muscle growth by fiber hypertrophy between 2 days and 3 months of age is documented. In total, 8,871 peptides could be identified (PEP value < 0.05) which could be assigned to 1,428 protein groups at a FDR < 0.01. All identified proteins are listed in Supplementary Table 1. In addition, ten proteins showing no significant abundance alteration between the four groups are listed in Supplementary Table 2. These proteins may be used as standards and loading controls for future quantitative experiments with biceps femoris samples of the pig DMD model.

Hierarchical clustering of LFQ values clearly separated the four groups, formed by genotype × age, with age being the higher level clustering factor (Fig. 1B). The numbers of significant (adjusted p-value < 0.05; log2-fold change > |0.6|) protein abundance changes are shown in Fig. 1C. In both, DMD and WT pigs, major age-dependent changes of the biceps femoris muscle proteome profile were observed, but the concordance of these changes between the two genotypes was limited (Fig. 1D). In both age groups, a large number of proteome differences between DMD and WT pigs were revealed, but the overlap of differentially abundant proteins was small (Fig. 1E). Differences in the abundance of selected proteins were verified by Western blot analysis (see Supplementary Fig. 1).

### Age-dependent proteome changes in WT and DMD pigs

In WT pigs, the marked muscle growth between day 2 and month 3 by fiber hypertrophy (Fig. 1A) was associated with abundance changes in a large number of proteins. The list of age-dependent proteome changes in WT muscle is given in Supplementary Tables 3a,b and the result of the DAVID^39^ analysis is shown in Supplementary Tables 3c–i. The most enriched ontology clusters of proteins with an age-related abundance increase were related to muscle contraction (17 proteins, Enrichment Score: 18.1), and glycolysis and/or glucose metabolic processes (14 proteins, Enrichment Score: 10.7). Proteins which showed an age-dependent decrease in abundance were mainly related to translation (50 proteins, Enrichment Score: 33.2), RNA processing (33 proteins, Enrichment Score: 5.0), and mRNA stability and posttranscriptional regulation of gene expression (14 proteins, Enrichment Score: 4.0).

A detailed list of age-dependent proteome changes in DMD muscle is shown in Supplementary Table 4a,b, results of the DAVID analysis are provided in Supplementary Table 4c–i. The main ontology clusters of proteins increasing in abundance with age were related to glycolysis or carbohydrate catabolic processes (14 proteins, Enrichment Score: 8.5) and muscle contraction/muscle system process (7 proteins, Enrichment Score: 4.0), whereas the major ontology categories of proteins with decreasing abundance were related to energy metabolism (60 proteins, Enrichment Score: 22.7), cellular respiration or TCA cycle (36 proteins, Enrichment Score: 15.3), and fatty acids or lipid metabolism (18 proteins, Enrichment Score: 4.4).

### Differentially abundant proteins in 2-day-old DMD vs. WT pigs

The comparison of the datasets from 2-day-old DMD pigs with the datasets of age-matched WT animals led to the detection of 53 proteins differing in abundance. DAVID analysis of the 15 proteins with increased abundance (Table 1) in DMD pigs led to 3 functional clusters, the most prominent being related to muscle development (5 proteins, Enrichment Score: 2.8) and cytoskeleton organization (4 proteins, Enrichment Score: 2.2). The corresponding results of DAVID GO annotation clustering are listed in Supplementary Table 5c,d.

**Figure 2:**
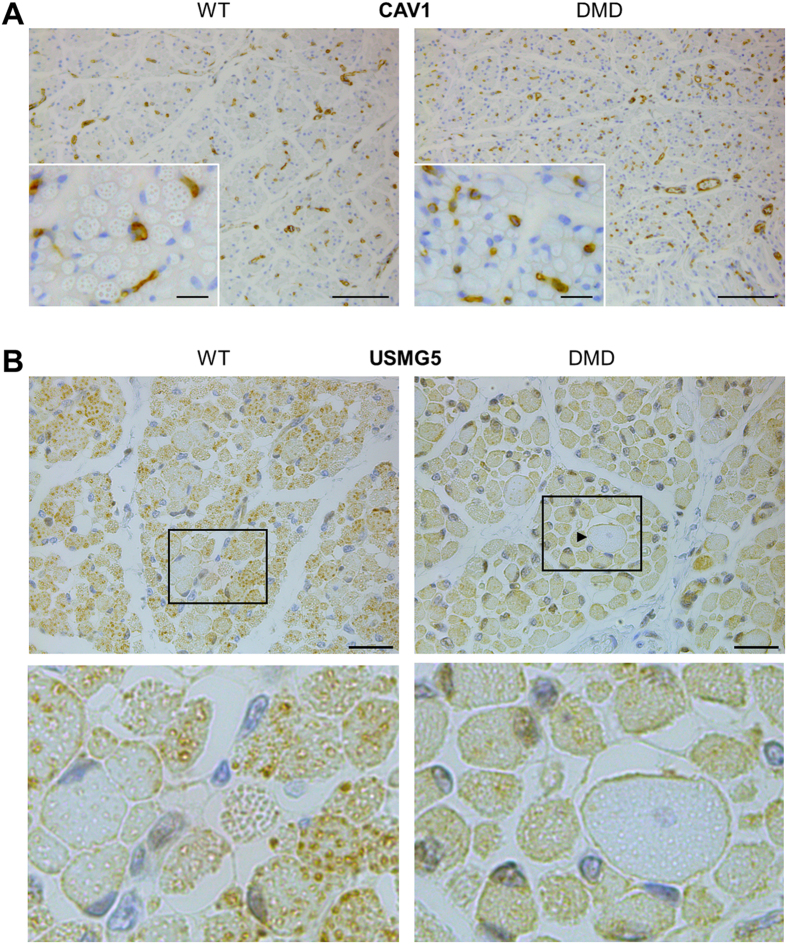
Localization of CAV1 and USMG5 in muscle sections from 2-day-old WT and DMD pigs. (**A**) CAV1 is predominantly detected in vascular endothelial cells. Bars represent 100 μm and 25 μm (insert). (**B**) In WT muscle, USMG5 staining labels small vesicular and granular structures in the cytoplasm of the majority of muscle fibers. In DMD muscle, the majority of fibers exhibit a more diffuse cytoplasmic staining. Some few muscle fibers show a similar staining pattern as seen in WT muscle cells. Further, large hypercontracted fibers (arrow head) of the DMD muscle with central nucleus exhibit a peripheral accentuated USMG5 staining at the sarcolemma. Lower panels represent magnifications of the framed areas of the upper panels. Bars represent 100 μm.

Two proteins with higher abundance in DMD muscle were selected for localization studies. Caveolin-1 (CAV1) was predominantly detected in vascular endothelial cells with apparently more stained cell profiles in sections of DMD compared to WT muscle (Fig. 2A). USMG5 [up-regulated during skeletal muscle growth 5 homolog (mouse)] was stained in small vesicular and granular structures in the cytoplasm of the majority of WT muscle fibers. In DMD muscle, most fibers exhibited a more diffuse cytoplasmic staining. Furthermore, large hypercontracted DMD muscle fibers with a central nucleus exhibited peripheral accentuated USMG5 staining at the sarcolemma (Fig. 2B).

DAVID enrichment analysis of the 38 proteins with lower abundance in DMD samples (Table 2) revealed two functional clusters, one related to translation (8 proteins, Enrichment Score: 6.2), the other to glycolysis (4 proteins, Enrichment Score: 3.1) (Supplementary Table 5e–g).

### Differentially abundant proteins in 3-month-old DMD vs. WT pigs

The quantitative analysis of the MS-data from 3-months-old DMD vs. corresponding WT pigs revealed 337 differently abundant proteins, of which 235 were more, and 102 less abundant in DMD pigs (Supplementary Table 6a,b). The DAVID analysis of the 235 proteins more abundant in DMD samples identified 17 protein clusters, the most prominent related to translation (25 proteins, 19 direct members of the ribosomal complex, Enrichment Score: 13.8). Further prominent clusters contained proteins related to extracellular structure/extracellular matrix organization (14 proteins, Enrichment Score: 5.7), actin/cytoskeleton organization (21 proteins, Enrichment Score: 5.2), and RNA processing (24 proteins, Enrichment Score: 5.0) (Supplementary Table 6c–g).

The DAVID analysis of all 102 proteins being less abundant in DMD muscle led to 13 protein clusters. The most prominent were related to glucose metabolism/glycolysis (18 proteins, Enrichment Score: 16.1), muscle/muscle contraction (19 proteins, Enrichment Score: 16.1), and energy metabolism/respiration/oxidative phosphorylation (34 proteins, Enrichment Score: 7.4) (Supplementary Table 6h–k).

**Figure 3:**
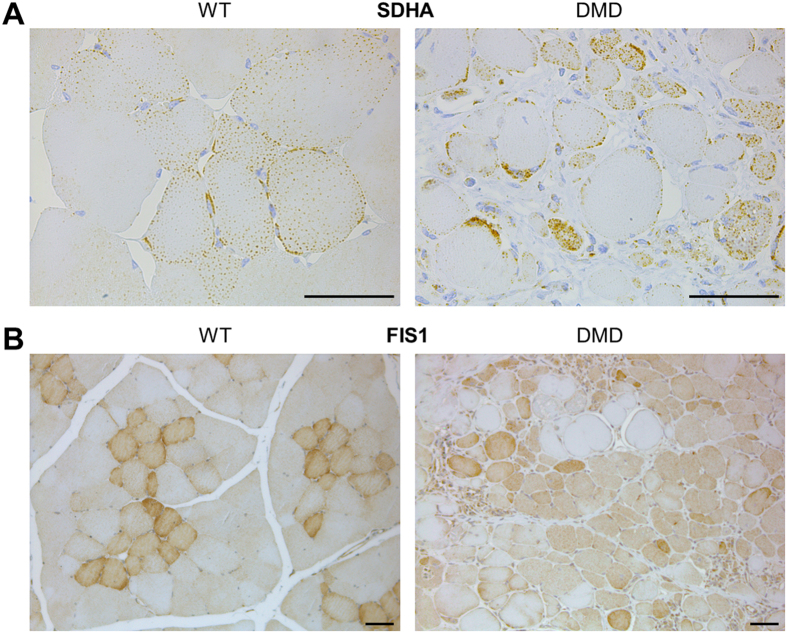
Immunohistochemical localization of mitochondrial proteins in 3-month-old WT and DMD muscle. (**A**) SDHA: In WT muscle, groups of myofibers exhibit granular cytoplasmic staining, some with peripheral accentuation. Adjacent fiber groups stain weakly or are not immunostained. DMD muscle shows very heterogeneous staining with a broad range of staining intensities and patterns between and within fibers. (**B**) FIS1: In WT, FIS1-immunostaining appears as a diffuse granular cytoplasmic pattern of different intensities. Within a muscle fascicle, predominantly central fibers show the strongest, adjacent fibers intermediate, and peripheral fibers the weakest staining intensity. In DMD muscle, a broad range of staining intensities of muscle fibers is visible without any consistent distribution pattern. Bars represent 50 μm.

**Figure 4:**
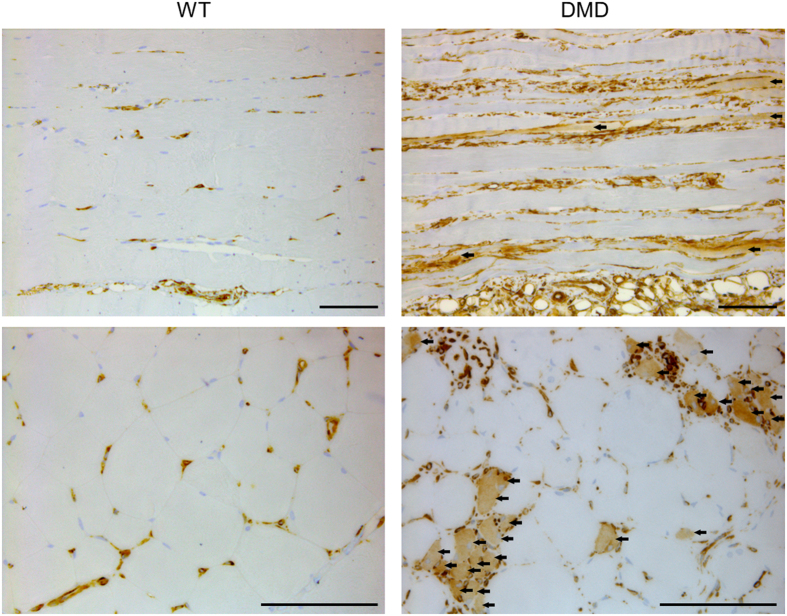
Immunohistochemical localization of VIM in 3-month-old WT and DMD muscle. VIM staining is strongly increased in DMD as compared to WT muscle. In wild-type muscle, VIM immunostaining is preferentially observed in small blood vessels. In DMD muscle, the increased interstitial tissue is strongly VIM-immunopositive. Further, several muscle fibers with centralized nuclei are VIM immunostained (marked by arrows). Upper panel: longitudinal sections; lower panel: cross-sections. Bars represent 100 μm.

Succinate dehydrogenase complex, subunit A (SDHA), which is an important catalytic subunit of succinate-ubiquinone oxidoreductase complex of the mitochondrial respiratory chain showed a 1.7-fold lower abundance in DMD samples and was selected for localization by immunohistochemistry (Fig. 3A). In muscle sections from 3-month-old WT pigs, clusters of myofibers were identified that exhibited granular cytoplasmic staining, some with peripheral sub-sarcolemmal accentuation. Adjacent fiber groups stained weakly or were apparently negative. Muscle sections from 3-month-old DMD pigs showed very heterogeneous staining with a broad range of staining intensities and patterns within and between fibers. In addition, we performed immunostaining of fission 1 (mitochondrial outer membrane) homolog (*S. cerevisiae*) (FIS1), an integral protein of the outer mitochondrial membrane, with higher abundance in 3-months-old DMD vs. WT muscle. In WT, FIS1 staining appeared as a granular diffuse cytoplasmic pattern of different intensities. Within a muscle fascicle, predominantly central fibers showed the strongest, adjacent fibers intermediate, and peripheral fibers the weakest staining intensity. In DMD muscle, a broad range of staining intensities of muscle fibers was present without any consistent distribution pattern (Fig. 3B). Furthermore, immunohistochemistry reflected the markedly increased levels of vimentin (VIM) detected by the LC-MS/MS analysis. Strong VIM immunostaining was observed in small groups of fibers with central nuclei, in some possibly regenerating muscle fibers, and in vascular and interstitial tissue, which was markedly increased in muscle from 3-month-old DMD pigs (Fig. 4).

### Fast and slow fiber types are differently affected by dystrophin deficiency in DMD pigs

**Figure 5:**
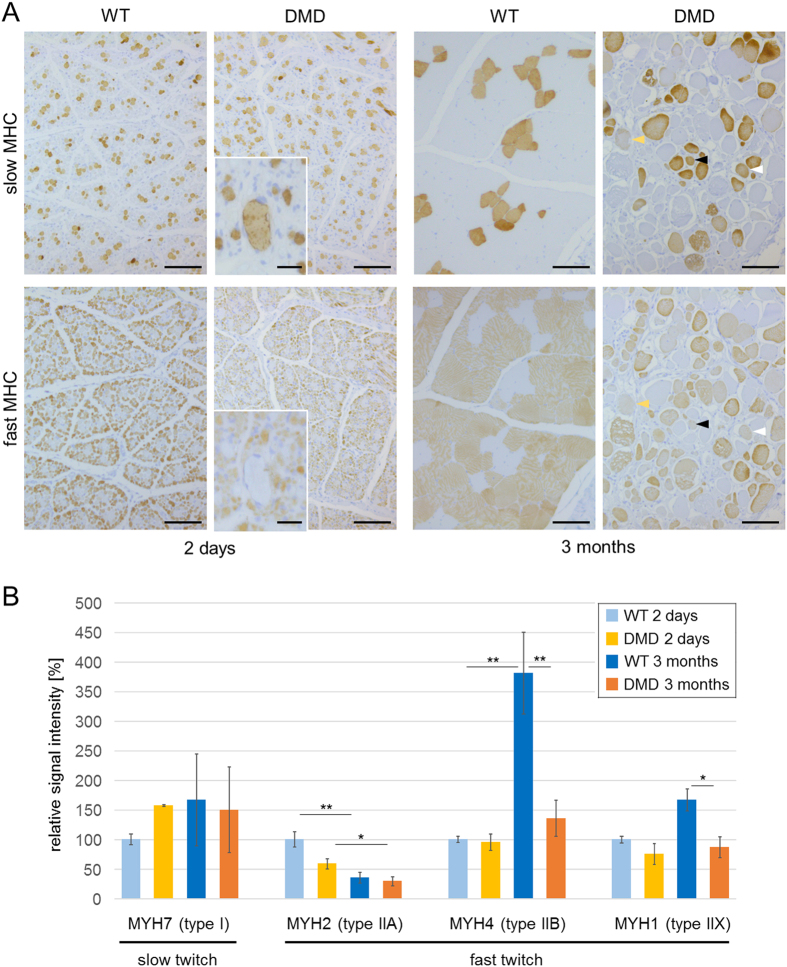
Characterization of muscle fiber types and abundances of fiber type specific myosin heavy chain proteins. (**A**) Immunohistochemical localization fast-twitch (MHCf) and slow-twitch (MHCs) muscle fibers in 2-day-old and 3-month-old WT and DMD muscle. In WT muscle, the fiber types exhibit an inverse staining pattern, which is more prominent in 3-month-old vs. 2-day-old muscle. In 3-month-old WT muscle, small groups of MHCs muscle fibers are embedded in a majority of MHCf muscle fibers. In DMD muscle, a heterogeneous pattern of MHCf and MHCs muscle fibers is observed with several fibers staining double positive (arrowheads). Bars represent 100 μm and 25 μm (insert). (**B**) Relative abundance of slow type I and fast type II myosin heavy chain proteins with fiber type specificity. The abundance of a particular myosin heavy chain protein in 2-day-old WT muscle was set to 100 percent. Lines indicate significant differences in abundance alterations (log2-fold change > |0.6|) between the corresponding groups. * corrected p-values < 0.05, ** corrected p-values < 0.01.

To address the question if specific muscle fiber types are preferentially affected in the DMD pig model, as suggested by abundance differences of specific myosin heavy chain proteins, we performed immunostaining using antibodies specific for fast-twitch (MHCf) and slow-twitch (MHCs) muscle fibers (Fig. 5A). In muscle sections from WT pigs of both age groups, the muscle fibers exhibited an inverse staining pattern for MHCf or MHCs. In 3-month-old wild-type muscle, small groups of MHCs fibers were found to be embedded in a majority of MHCf fibers. In DMD muscle, a heterogeneous pattern of MHCf and MHCs muscle fibers was observed with several fibers staining double positive (arrow heads). Our proteomics data for myosin heavy chains allowed further specification of affected fiber types (Fig. 5B). While there was no significant difference in MYH7 (specific for slow twitch type I fibers) among the groups, MYH2 (specific for fast twitch type IIA fibers) decreased significantly with age in both WT and DMD muscle. The most prominent differences were seen for MYH4 (specific for fast twitch type IIB fibers), which was 4-fold more abundant in 3-month-old vs. 2-day-old WT pigs, whereas in DMD pigs no significant age-related increase was observed, resulting in a markedly reduced MYH4 level in 3-month-old DMD vs. WT muscle. The same was observed for MYH1, a marker of fast twitch type IIX fibers.

## Discussion

Proteome profiling of different stages of diseased vs. healthy tissues holds the potential to gain new insights into disease mechanisms and to discover targets and molecular readouts for therapeutic interventions. Due to unavoidable restrictions of systematic muscle sampling in human patients, this approach is not feasible for Duchenne muscular dystrophy patients. Whereas several recent proteome studies of human serum^40^,^41^,^42^,^43^ or urine samples^44^ reported biomarkers for DMD, systematic proteome studies of dystrophic skeletal muscle are only available for animal models of DMD, primarily the *mdx* mouse, which has, however, limitations in resemblance of human DMD pathology^31^. Large animal models showing strong phenotypes are the GRMD model^33^,^34^ and the DMD pig^38^, which was used for this study. Since one of our goals was to provide novel molecular readouts for future treatment trials, we chose the biceps femoris muscle of which corresponding samples can be accessed by biopsy without sacrificing the animal. In addition, quantitative morphological parameters indicative of the severity of muscular dystrophy were available for these samples^38^. As metabolic labeling like SILAC is not applicable for large animal models like the pig, we chose the label free quantification (LFQ) approach^45^,^46^. In order to focus on candidates being quickly assessable with routine LC-MS methods, we decided to keep the proteomics workflow as simple as possible without chemical labeling. Instead of pre-fractionation steps, which could prevent reliable LFQ-based protein quantification, we used long chromatographic gradients (440 min) combined with long separation columns (50 cm, 2 μm beads) to diminish under-sampling. To ensure the reproducibility of our label free quantification experiments, we carefully controlled retention time stability between each run. Although proteomics of skeletal muscle is particularly challenging due to the high abundance of several proteins like myosins, troponins and tropomyosins, we were able to identify 1,428 proteins at a false discovery rate of <1% which is markedly more than in previous studies investigating non-rodent animal models like the GRMD dog model^34^. The entire dataset was submitted to ProteomeXchange^47^ with the dataset identifier PXD002918 and to the Pig PeptideAtlas^48^. In order to verify the reliability of our datasets, we performed hierarchical clustering of the proteome profiles leading to a heat map perfectly reflecting the 4 different groups defined by our 2 genotypes × 2 ages design. Age was the highest clustering factor, indicating major proteome changes in growing skeletal muscle between 2 days and 3 months of age. Importantly, our dataset confirms the previously shown absence of dystrophin in samples from DMD pigs and the up-regulation of utrophin in 3-month-old DMD vs. age-matched WT animals^38^ (see Supplementary Tables 5b and 6b).

The comparison of protein abundances between 2-day-old DMD and WT animals reveals early derangements of DMD muscle. Among the proteins more abundant in muscle of 2-day-old DMD animals was caveolin-1 (CAV1), an integral membrane protein with preferred location in caveolae. Proposed functions include lipid transport, membrane traffic, and cell signaling (reviewed in ref. 49). In masticatory muscles of *mdx* mice, CAV1 was associated with blood vessels in areas with regenerating muscle fibers^50^. In vascular endothelial cells, CAV1 was shown to form a complex with dystrophin and endothelial NO synthase^51^. We localized CAV1 in blood vessels of 2-day-old WT and DMD pigs, with an apparent trend of more stained cells in the latter group (see Fig. 2A).

Another protein with increased abundance in 2-day-old DMD vs. WT pigs was Xin actin-binding repeat-containing protein 1 (XIRP1), a multi-adaptor protein which plays important roles in the assembly and repair of myofibrils and is capable of binding filamin C (FLNC) and the dystrophin-binding protein aciculin (PGM5)^52^. Of interest, FLNC abundance was also increased in muscle samples from 2-day-old DMD vs. WT pigs. The same is true for titin-cap (TCAP, previously termed telethonin), a muscle-specific titin-capping protein which provides structural support to the sarcomere by linking the N-terminus of titin to other z-disc proteins. Collectively, these findings suggest that up-regulation of proteins maintaining the structure of sarcomeres is an early compensatory response to dystrophin deficiency.

Up-regulated during skeletal muscle growth 5 homolog (mouse) (USMG5) was increased in abundance in 2-day-old DMD pigs. USMG5 is a small subunit of the mitochondrial ATP synthase and of the lysosomal V-ATPase. Immunostaining of USMG5 in rat skeletal muscle revealed a higher abundance in highly oxidative than in less oxidative or glycolytic muscle fibers^53^. USMG5 localization in muscle fibers from 2-day-old WT and DMD pigs revealed distinctly different staining patterns, with a clear association of USMG5 to granular and vesicular structures in the cytoplasm of WT muscle cells, and a more diffuse USMG5 staining in most DMD muscle fibers (see Fig. 2B). Interestingly, a study investigating determinants of disease severity in DMD found 4.7-fold increased *USMG5* mRNA levels in muscle biopsies from patients with late vs. early loss of ambulation^54^. Thus, the increase of USMG5 in muscle samples from 2-day-old DMD pigs may represent an early rescue mechanism.

The quantitative analysis of the MS-data from 3-months-old DMD vs. corresponding WT pigs revealed differently abundant proteins from a variety of functional classes.

Human DMD is associated with an increase of connective tissue (fibrosis) as a reparative response to muscle damage and injury^55^, resulting in an accumulation of extracellular matrix proteins. Similarly, the skeletal muscle of the DMD pig model showed an increase of fibrotic tissue ongoing with an increase of transcripts related to proteins of the extracellular matrix^38^. This is in line with our observation that a variety of extracellular matrix proteins were found to be more abundant in the samples of 3-month-old DMD pigs. For instance, we found the proteins collagen type VI alpha 1 (COL6A1, 1.9-fold) and collagen type VI alpha 3 (COL6A3, 2.6-fold) significantly enriched in the muscle of 3-months-old DMD pigs. Both proteins are subunits of collagen VI which is an important component of the muscular extracellular matrix (ECM) and mainly provided by interstitial muscular fibroblasts^56^. An increase of COL6A1 was previously detected in the diaphragm of *mdx* mice during aging^28^, in diaphragm samples of *mdx*^20^ and *mdx-4cv*^57^ mice, as well as in skeletal muscle samples of *mdx* compared to WT animals^23^,^26^. The importance of muscle collagen VI is reflected by diseases like the Bethlem myopathy and the Ullrich congenital muscular dystrophy caused by mutations of the *COL6* genes. Furthermore, it could be demonstrated that collagen VI is a component of the satellite cell niche and important for satellite cell self-renewal and muscle regeneration^58^. The increased abundance of collagen VI in the proteome of the DMD pig may therefore reflect ongoing muscle regeneration. In addition, we found increased levels of collagen type XII alpha 1 (COL12A1, 2.6-fold) in muscle of 3-month-old DMD pigs. Interestingly, a mutation of this gene was also found to be associated with a Bethlem myopathy-like phenotype of patients with absent mutations in the collagen VI genes^59^.

Additionally, several intermediate filament (IF) proteins were more abundant in muscle tissue of 3-month-old DMD pigs as compared to the WT animals. Among them were nestin (33-fold), vimentin (9-fold), and desmin (2.5-fold). While nestin is a type IV IF, desmin and vimentin belong to the type III family of IF proteins. During regeneration of injured adult rat muscle, nestin was found to be present in myoblasts and myotubes and to form filaments with vimentin and desmin^60^. For vimentin, abundance alterations were already observed in a variety of proteomics studies analyzing skeletal muscle of *mdx* mice^20^,^21^,^23^,^26^,^30^ and from the GRMD model^34^. Additionally, elevated desmin abundances were observed in skeletal muscle^20^,^21^ and diaphragm^61^ of *mdx* and in diaphragm of *mdx-4cv* mice^57^. Increased vimentin and desmin abundance in dystrophic muscles was discussed as a compensatory mechanism supporting the structural organization of the sarcomere^62^. Vimentin localization revealed that the marked increase of vimentin in muscle samples from 3-month-old DMD pigs was mainly due to a large proportion of vimentin positive interstitial tissue (see Fig. 4).

In addition to IF proteins, the extracellular matrix protein tenascin C (TNC), known to be involved in muscle repair^63^, was more abundant in muscle samples from 3-month-old DMD pigs. In *mdx* mouse muscle, TNC staining was prominent in degenerating/regenerating areas, but absent from undegenerated muscle. TNC was also detected in muscle from DMD boys and DMD dog models, and it was suggested that TNC expression is stimulated by muscle cell degeneration and remains high unless successful regeneration occurs^64^. Taken together, the increased abundance of intermediate filament proteins and of tenascin C in the samples of 3-month-old DMD pig muscle is in line with findings made in other DMD models and may reflect ongoing regeneration of damaged muscle fibers.

Strikingly, many proteins related to energy metabolism were less abundant in muscle samples from 3-month-old DMD pigs compared to age-matched WT controls. Remarkably, reduced abundance of proteins from all respiratory chain complexes was observed pointing to an impaired oxidative energy production in 3-month-old DMD pig muscles. In addition, SDHA showed a deranged staining pattern in DMD compared to WT muscle (Fig. 3A). This observation in our pig model reflects results of previous proteomics studies analyzing skeletal muscle of *mdx* mice^21^,^22^ and GRMD dogs^34^. The decrease of oxidative phosphorylation enzymes may be explained by the relative reduction of functional muscle tissue and an increase of interstitial fibrosis revealed by our morphological findings in DMD pig muscle.

A recent study of *mdx* mouse muscle showed abnormal localization of subsarcolemmal mitochondria and impaired ATP-generating capacity^65^. A further study investigating isolated muscle mitochondria of 12-week-old *mdx* and healthy control mice suggested Complex I insufficiency as a reason for impaired mitochondrial ATP production^66^. Since the DMD pig shows more severe symptoms than the *mdx* mouse and a particularly high number of respiratory chain proteins decreased in abundance, our findings suggest the pig model to be especially suitable to further study DMD related mitochondrial dysfunction.

Beside proteins of the respiratory chain complexes, mitochondrial fission 1 protein (FIS1) is increased in abundance in DMD pig muscle. Mitochondrial fission and fusion are crucial processes determining morphology and dynamics of the mitochondrial network. Furthermore, fission followed by selective fusion segregates dysfunctional mitochondria and allows their removal by autophagy^67^. We found FIS1 more abundant in 3-month-old DMD vs. WT pigs. FIS1 promotes mitochondrial and peroxisomal fission by recruiting the dynamin-related protein, Drp1 (DMN1L)^68^. Besides, FIS1 is supposed to be involved in the fragmentation of the mitochondrial network and its perinuclear clustering^69^. Interestingly, a recent study found increased levels of Drp1 and mitofusin 2 (MFN2, important for mitochondrial fusion) in extensor digitorum longus muscle samples of a utrophin-dystrophin deficient (DKO) mouse model^70^. The authors speculated that increased mitochondrial fission-fusion rates may compensate an elevated energy production in response to a higher demand due to muscle damage and oxidative inefficiency. The localization of FIS1 in muscle sections from 3-month-old DMD pigs (Fig. 3B) was in line with this hypothesis. Their muscle fibers showed a broad range of staining intensities without any defined pattern, whereas in WT muscle a clear gradient of three staining intensities was apparent in each muscle fascicle, with the strongest staining of fibers in the center and the weakest in the periphery.

Strikingly, beside proteins related to energy production, several proteins playing an important role for muscle contraction are altered in abundance in the 3-month-old DMD pigs. Mammalian skeletal muscle tissue consists of different types of muscle fibers. The so called “slow” type I muscle fibers contract slower and with smaller force, but fatigue slower as compared to other muscle fiber types^71^,^72^. In contrast, so called “fast” type II muscle fibers contract faster and with higher force^73^, but fatigue earlier^74^. According to molecular properties of their myosin heavy chain (MYH), type II fibers can be subdivided in type IIa fibers containing MYH2 (also known as MyHC-IIa), type IIb fibers with MYH4 (also known as MyHC-IIb), and type IIx fibers with MYH1 (also known as MyHC-2x)^75^. The characteristic myosin heavy chain for type I fibers is MYH7 (also referred to as MyHC-slow). Interestingly, the abundances of these characteristic MYH proteins, were differently altered in the 3-month-old DMD animals. As shown in Fig. 5, the abundance of MYH7 and MYH2, is not affected by the lack of dystrophin. In contrast, MYH1 and MYH4 were strongly decreased in abundance in DMD muscle. These findings are in line with results from a gastrocnemius muscle proteome study of 6-week-old *mdx* mice^26^ and with those from human DMD patients exhibiting a fast-to-slow fiber type shift with strong decrease of fast glycolytic muscle fibers^76^,^77^. This shift might be caused by preferential susceptibility of fast fiber types (especially type IIb) to degeneration in DMD^76^. Since in DMD pig muscle numerous fibers stained positive for both MHCs and MHCf, the increased proportion of slow fibers in DMD muscle may in part also be a result of fiber type shifting.

## Conclusions

Our proteome study of the first pig model of DMD revealed a large number of stage specific proteome changes, many of which are in line with findings of previous proteome studies with other well established DMD animal models and thus reflect the relevance of our data and animal model. Additionally, we provide new insights into the nature and time course of molecular derangements of dystrophic muscle. Especially the early alterations of the muscle proteome may be used for evaluation of efficacy of new therapies, such as exon skipping or gene editing, thereby complementing clinical and morphological readouts. Promising results of these novel therapies have been achieved in the mouse model^8^,^9^,^10^,^11^ and in preclinical studies performed using the GRMD dog model (reviewed in ref. 5). The major advantage of the DMD pig model over the GRMD model is the type of mutation which corresponds to a frequent human DMD mutation. This is important for testing targeted therapies, such as exon skipping^8^ or CRISPR/Cas mediated exon deletion^9^,^10^,^11^. Since the DMD pig shows major pathological hallmarks of human DMD already at an earlier stage, targeted therapies such as exon skipping can be started early after birth to test if the rapid progression of the disease can be – at least partially – prevented. In a large animal model of human disease even intrauterine treatment strategies could be tested. Further evaluation and refinement of these novel therapy approaches in DMD pigs may aid their translation into clinical application.

## Methods

### Animal model and tissue samples

All animal experiments were carried out in accordance with the German Animal Welfare Act and were approved by the responsible animal welfare authority (District Government of Upper Bavaria, Reference Number 55.2-1-54-2531-86-10). DMD pigs were generated as described before^38^. DMD piglets and age-matched WT controls were euthanized at 2 days and approximately 3 months of age (n = 3 per genotype and age). For this study we focused on samples of biceps femoris, since for future treatment trials corresponding biopsies can be easily performed without sacrificing the animal. Ca. 100 mg of biceps femoris were shock frozen on dry ice, and stored at −80 °C until further analysis. Additional samples were fixed in neutrally buffered formaldehyde solution (4%) for 24 hours, routinely processed and embedded in paraffin or in plastic [glycol methacrylate and methyl methacrylate (GMA/MMA)]^38^.

### Sample processing

Per mg frozen tissue 15 μl of 8 M urea/0.4 M NH_4_HCO_3_ was added. Tissues were lysed using a homogenizer (ART-MicraD8, ART Prozess- & Labortechnik, Müllheim, Germany) at a speed of 23,500 rpm for 30 sec and centrifuged through QIA-Shredder devices (Qiagen, Hilden, Germany). Protein concentrations were determined by Bradford assay^78^ and adjusted with 8 M urea/0.4 M NH_4_HCO_3_ to 1 mg/ml. 100 μg protein was reduced with DTE at a concentration of 4 mM for 30 min and cysteine residues were blocked with iodoacetamide (final concentration 8 mM) for 30 min in the dark. After dilution with water to a concentration of 1 M urea, 2 μg porcine trypsin (Promega, Madison, WI, USA) was added and incubated overnight at 37 °C.

### Mass spectrometry

LC-MS/MS was performed on an EASY-nLC 1000 chromatography system (Thermo Scientific, Waltham, MA, USA) connected to an Orbitrap XL instrument (Thermo Scientific). Five μg of peptides diluted in 0.1% formic acid (FA) were transferred on a trap column (PepMap100 C18, 75 μm × 2 cm, 3 μm particles, Thermo Scientific) and separated at a flow rate of 200 nL/min (Column: PepMap RSLC C18, 75 μm × 50 cm, 2 μm particles, Thermo Scientific) using consecutive linear gradients from 2% to 25% solvent B (0.1% formic acid, 100% ACN) in 320 min and from 25% to 50% solvent B in 120 min. For data acquisition, a top five data dependent CID method was used. Data were transferred to ProteomeXchange^47^ with the dataset identifier PXD002918.

### Bioinformatics

MS data were processed, using MaxQuant V1.5.1^45^ and the *Sus scrofa* subset of the UniProt database. For identification, the following parameters were used: i) Enzyme: Trypsin; ii) Mass tolerance precursor: 10 ppm; iii) Mass tolerance MS/MS: 0.8 Da; iv) Fixed modification: Carbamidomethylation of cysteine; v) Variable modifications: acetylation of protein N-terminus and oxidized methionine. FDRs at the peptide and protein level were set to 1%. For label-free quantification (LFQ)^46^ the match between runs option was enabled. Hierarchical clustering was performed with the Perseus module of MaxQuant. In case proteins were detected in all replicates of one group, but in no replicate of the other group, the MaxQuant imputation feature was used to allow a statistical evaluation. Further statistics was done with R^79^. A 2-way ANOVA with age and genotype as fixed effects was performed, followed by Tukey Honest Significant Differences post-hoc test to verify differences between the groups. To correct the results of the 2-way ANOVA for multiple testing, we chose a FDR-based approach and calculated the q-value using the R package “qvalue”. This approach avoids false positive results while offering a more liberal correction criterion compared to other methods^80^. For the DAVID analysis as well as for the discussion section, we exclusively used proteins showing an abundance alteration of a log2fold >│0.6│ and a significance level of <0.05 after correction for multiple testing. To facilitate a meta-analysis addressing also less prominent abundance alterations, the quantitative values for all identified proteins are listed in Supplementary Table 1. For functional annotation clustering, the DAVID online platform^39^ was used.

### Western blot analysis

Protein samples (n = 3 per group, 15 μg per sample) were separated on a 5% stacking gel (62.5 mM Tris-HCl pH 6.8, 5% acrylamide/bis-acrylamide (37.5:1), 0.1% SDS, 0.125% APS, 0.055% TEMED) and a 12% separation gel (0.375 M Tris-HCl pH 8.8, 12% acrylamide/bisacrylamide (37.5:1), 0.1% SDS, 0.05% APS, 0.05% TEMED) with a mini-Protean Tetra cell (BioRad). Separated proteins were transferred onto PVDF membrane (0.45 μm, IPVH00010, Millipore) for 30 min at 1.0 A/25 V. Equal loading was assessed by Ponceau S staining. The blots were blocked (5% non-fat dry milk in Tris-buffered saline with 0.1% Tween 20) for 1 h and probed overnight at 4 °C with the primary antibodies listed in Supplementary Table 7. Detection was performed with horseradish peroxidase-conjugated polyclonal goat anti-rabbit antibody (1:2000, no. 7074, Cell Signaling; 1 h at RT), enhanced chemiluminescence substrate (32106, Pierce/Thermo Scientific) and Amersham Hyperfilm ECL films (28906837, GE Healthcare). Western blot bands were quantitatively analyzed by ImageJ 1.50i^81^. Total protein normalization was performed on densitometric measurements of Ponceau S staining along the complete separation range of each lane. Unpaired t-test was calculated in Excel.

### Morphological analysis and immunohistochemistry

Histological analyses of biceps femoris muscle samples were performed as described previously^38^. Plastic sections (glycol methacrylate/methyl methacrylate, GMA/MMA^82^) were stained with hematoxylin and eosin (H&E) to show the degree of dystrophy of biceps femoris muscle from 2-day-old and 3-month-old DMD pigs as compared to age-matched WT controls. Immunohistochemistry was performed on paraffin sections (5–7 μm), using the primary and secondary antibodies listed in Supplementary Table 7, VECTASTAIN Elite ABC-Peroxidase Kit when using biotinylated secondary antibody, and 3,3′-diaminobenzidine tetrahydrochloride dihydrate (DAB). Nuclear counterstaining was done with hemalum.

## Additional Information

**How to cite this article**: Fröhlich, T. *et al.* Progressive muscle proteome changes in a clinically relevant pig model of Duchenne muscular dystrophy. *Sci. Rep.* **6**, 33362; doi: 10.1038/srep33362 (2016).

## Supplementary Material

Supplementary Information

Supplementary Table 1

Supplementary Table 2

Supplementary Table 3

Supplementary Table 4

Supplementary Table 5

Supplementary Table 6

## Figures and Tables

**Table 1:** List of proteins more abundant in muscle of 2-day-old DMD pigs compared to age matched WT pigs.

Accession	Entry name	Protein	Gene	Log2-fold change	adjusted p-value
F1RFD4	F1RFD4_PIG	Uncharacterized protein [up-regulated during skeletal muscle growth 5 homolog (mouse)]	USMG5	in DMD only	0.00027 *
Q6RVA9	CAV1_PIG	Caveolin-1	CAV1	in DMD only	0.00001 *
Q6QAT0	RL32_PIG	60S ribosomal protein L32	RPL32	in DMD only	0.00099 *
F1SJM0	F1SJM0_PIG	Uncharacterized protein [pentraxin 3]	PTX3	in DMD only	0.01351 *
F1SJR2	F1SJR2_PIG	Uncharacterized protein [xin actin-binding repeat-containing protein 1]	XIRP1	2.15	0.01749
A4GR69	A4GR69_PIG	Telethonin (uncharacterized protein)	TCAP	1.77	0.00109
Q53DY7	Q53DY7_PIG	Histone H1.3-like protein (fragment)		0.97	0.03228
I3L6Q5	I3L6Q5_PIG	Uncharacterized protein [histone cluster 1, H1b]	HIST1H1B	0.84	0.01500
F1RWW4	F1RWW4_PIG	Uncharacterized protein [PDZ and LIM domain 5]	PDLIM5	0.83	0.00137
Q6QGC0	PDLI3_PIG	PDZ and LIM domain protein 3 (actinin-associated LIM protein) (alpha-actinin-2-associated LIM protein)	PDLIM3	0.79	0.02916
F2Z4Y1	F2Z4Y1_PIG	Uncharacterized protein [tyrosine 3-monooxygenase/tryptophan 5-monooxygenase activation protein, eta]	YWHAH	0.71	0.01067
F1SMN5	F1SMN5_PIG	Uncharacterized protein [filamin C, gamma]	FLNC	0.69	0.04389
F1SS66	F1SS66_PIG	Uncharacterized protein [myosin, heavy chain 13, skeletal muscle]	MYH13	0.68	0.01007
P82460	THIO_PIG	Thioredoxin (Trx)	TXN	0.67	0.00124
P62802	H4_PIG	Histone H4		0.65	0.03889

Values with log2-fold change >│0.6│ and p < 0.05 were considered as significant. In addition, proteins identified in muscle of 2-day-old DMD pigs but not in 2-day-old WT pigs are listed. To calculate p-values tagged with *, missing values were imputed.

**Table 2:** List of proteins less abundant in muscle of 2-day-old DMD pigs compared to age matched WT pigs.

Accession	Entry name	Protein	Gene	Log2-fold change	adjusted p-value
A1XQV5	A1XQV5_PIG	Fast skeletal muscle troponin C (troponin C, skeletal muscle)	TNNC2	−0.62	0.02272
L7PBE6	L7PBE6_PIG	T-complex protein 1 subunit epsilon	CCT5	−0.62	0.02998
D0G0C8	D0G0C8_PIG	Chaperonin containing TCP1, subunit 2 (Beta) (uncharacterized protein)	CCT2	−0.64	0.04207
A1XQU5	RL27_PIG	60S ribosomal protein L27	RPL27	−0.69	0.00102
I3LII3	I3LII3_PIG	eukaryotic translation elongation factor 2 [EEF2]	EEF2	−0.69	0.00262
F1RZ28	F1RZ28_PIG	Uncharacterized protein [ribosomal protein S10]	RPS10	−0.71	0.01468
F1RQ91	F1RQ91_PIG	40S ribosomal protein S4	RPS4	−0.74	0.02382
Q29092	ENPL_PIG	Endoplasmin (94 kDa glucose-regulated protein) (GRP-94)	HSP90B1	−0.76	0.00275
F1S9C9	F1S9C9_PIG	Proteasome subunit beta type (EC 3.4.25.1)	LOC100155139	−0.76	0.03231
P62844	RS15_PIG	40S ribosomal protein S15 (RIG protein)	RPS15 RIG	−0.76	0.04926
F1SHL9	F1SHL9_PIG	Pyruvate kinase (EC 2.7.1.40) (fragment)	PKM	−0.81	0.00137
I3LHF0	I3LHF0_PIG	Uncharacterized protein [eukaryotic translation elongation factor 1 delta]	EEF1D	−0.81	0.02079
F1RFY2	F1RFY2_PIG	Beta-enolase	ENO3	−0.84	0.01203
I3LJ87	I3LJ87_PIG	Uncharacterized protein [ribosomal protein SA]	RPS2	−0.86	0.02618
F1SHD6	F1SHD6_PIG	Uncharacterized protein [eukaryotic translation elongation factor 1 beta 2]	EEF1B2	−0.89	0.00313
F1SIT7	F1SIT7_PIG	60S acidic ribosomal protein P1-like	LOC100523874	−0.92	0.01276
P00355	G3P_PIG	Glyceraldehyde-3-phosphate dehydrogenase (GAPDH)	GAPDH	−0.94	0.00132
Q29387	EF1G_PIG	Elongation factor 1-gamma (EF-1-gamma) (eEF-1B gamma) (fragment)	EEF1G	−0.94	0.01521
I3L5B2	I3L5B2_PIG	Uncharacterized protein [ribosomal protein S7]	RPS7	−0.94	0.02299
I3LAK5	I3LAK5_PIG	Uncharacterized protein [fragile X mental retardation, autosomal homolog 1]	FXR1	−0.94	0.04305
Q1HL06	Q1HL06_PIG	Muscle 6-phosphofructokinase (fragment)	PFMK	−1.00	0.00844
B9W5V0	B9W5V0_PIG	Elongation factor 1-alpha	EEF1A2	−1.00	0.01373
F1S814	F1S814_PIG	Uncharacterized protein [phosphoglucomutase 1]	PGM1	−1.03	0.00673
F1SUM3	F1SUM3_PIG	Uncharacterized protein (fragment) [serpin peptidase inhibitor, clade H (heat shock protein 47), member 1]	SERPINH1	−1.06	0.00021
P61288	TCTP_PIG	Translationally-controlled tumor protein (TCTP)	TPT1	−1.12	0.01265
P00339	LDHA_PIG	L-lactate dehydrogenase A chain (LDH-A) (EC 1.1.1.27) (LDH muscle subunit) (LDH-M)	LDHA	−1.18	0.02031
F1RUN2	F1RUN2_PIG	Serum albumin	ALB	−1.84	0.019
P01846	LAC_PIG	Ig lambda chain C region		−3.64	0.0036
I3LAQ0	I3LAQ0_PIG	Uncharacterized protein [homology to immunoglobulin kappa variable region, partial]	LOC100737103	−3.64	0.00985
L8B0U3	L8B0U3_PIG	IgG heavy chain	IGHG	−3.84	0.03484
L8B0R9	L8B0R9_PIG	IgG heavy chain	IGHG	−4.32	0.00909
F1RRY6	F1RRY6_PIG	Uncharacterized protein (fragment) [prostaglandin E synthase 2]	PTGES2	in WT only	0.00328 *
I3LR43	I3LR43_PIG	Uncharacterized protein [peptidase D]	PEPD	in WT only	0.00827 *
I3LQ17	I3LQ17_PIG	Uncharacterized protein [homology to alpha-2-macroglobulin]		in WT only	0.03762 *
F1RUQ0	F1RUQ0_PIG	Uncharacterized protein [immunoglobulin J polypeptide, linker protein for immunoglobulin alpha and mu polypeptides]	IGJ	in WT only	0.00854 *
I3LHD8	I3LHD8_PIG	Uncharacterized protein [coiled-coil domain containing 85C]	LOC100512614	in WT only	0.00038 *
L8B0W9	L8B0W9_PIG	IgG heavy chain	IGHG	in WT only	0.02316 *
Q5GN48	DMD_PIG	Dystrophin	DMD	in WT only	<0.00001 *

Values with log2-fold change >│0.6│ and p < 0.05 were considered as significant. In addition, proteins identified in muscle of 2-day-old WT but not in 2-day-old-DMD pigs are listed. To calculate p-values tagged with * missing values were imputed.
