# Supplementary material for: Progressive muscle proteome changes in a clinically relevant pig model of Duchenne muscular dystrophy
Source: Sci Rep. 2016 Sep 16;6:33362. doi: 10.1038/srep33362 (PMC5025886; doi:10.1038/srep33362)
Supplement: Supplementary Information [file srep33362-s1.pdf]

Progressive muscle proteome changes in a clinically relevant pig model of Duchenne muscular dystrophy

Thomas Fröhlich[1], Elisabeth Kemter[2], Florian Flenkenthaler[1], Nikolai Klymiuk[2], Kathrin A. Otte[1], Andreas Blutke[3], Sabine Krause[4], Maggie C. Walter[4], Rüdiger Wanke[3], Eckhard Wolf[1,2,], Georg J. Arnold[1,]**

[1]Laboratory for Functional Genome Analysis (*LAFUGA*), Gene Center, LMU Munich, Feodor-Lynen-Str. 25, D-81377 Munich, Germany

[2]Chair for Molecular Animal Breeding and Biotechnology, Gene Center and Department of Veterinary Sciences, LMU Munich, Feodor-Lynen-Str. 25, D-81377 Munich, Germany

[3]Institute of Veterinary Pathology, Centre for Clinical Veterinary Medicine, LMU Munich, Veterinärstr. 13, D-80539 Munich, Germany

[4]Friedrich-Baur-Institute, Department of Neurology, LMU Munich, Marchioninistr. 17, D-81377 Munich, Germany

*equal last author contribution

Corresponding Authors

Eckhard Wolf (ewolf@genzentrum.lmu.de)

Phone: +49-89-2180-76800; Fax: +49-89-2180-76849

Georg J. Arnold (arnold@genzentrum.lmu.de)

Phone: +49-89-2180-76825; Fax: +49-89-2180-76848

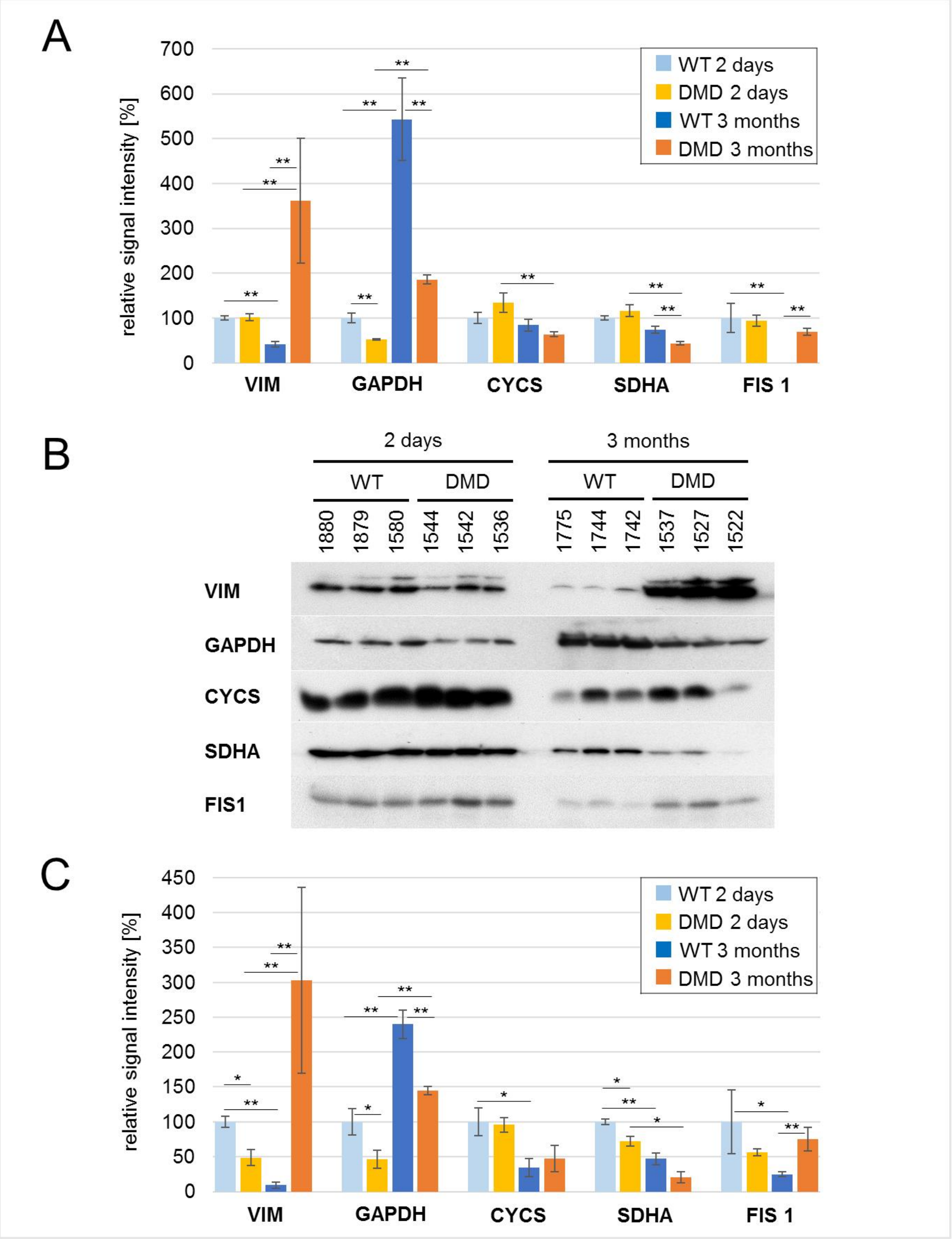

Supplementary Figure 1. Comparison of results obtained by mass spectrometry based quantification with corresponding Western immunoblots

A) Relative abundance of vimentin (VIM), glyceraldehyde-3-phosphate dehydrogenase (GAPDH), cytochrome c (CYCS), succinate dehydrogenase complex subunit A (SDHA) and mitochondrial fission 1 protein (FIS1) in biceps femoris muscle tissue samples from the four animal groups. The abundance of the proteins in 2-day-old WT muscle was set to 100 percent. Error bars represent standard deviations within the group of animals; lines and ** indicate significant differences in abundance alterations (log2-fold change > |0.6|, corrected p-value < 0.01) between the corresponding groups.

B) Western blot analysis of VIM, GAPDH, CYCS, SDHA and FIS1 in biceps femoris muscle tissue samples of WT and DMD pigs at 2 days and 3 months of age.

C) Quantitative evaluation of the Western blot analysis of VIM, GAPDH, CYCS, SDHA and FIS1 in biceps femoris muscle tissue samples of WT and DMD pigs at 2 days and 3 months of age. The abundance of the proteins in 2-day-old WT muscle was set to 100 percent. Error bars represent standard deviations within the group of animals; * indicate p-values < 0.05 and ** p-values < 0.01.

Supplementary Table 7: Antibodies used for Western blot and immunohistochemistry

Target	Primary Antibodies	Dilution Western Blot	Dilution Immunohistochemistry
CAV1	rabbit monoclonal antibody, D46G3, no. 3267, Cell Signaling	-	1:800
CYCS	rabbit monoclonal antibody, 136F3, no. 4280, Cell Signaling	1:1000	-
FIS1	rabbit polyclonal antibody no. GTX111010, GeneTex	1:10000	1:2200
GAPDH	rabbit monoclonal antibody , no. 2118, Cell Signaling	1:1000	-
Myosin Heavy Chain fast	mouse monoclonal antibody, no. NCL-MHCf, Novocastra Laboratories Ltd	-	1:400
Myosin Heavy Chain slow	mouse monoclonal antibody, no. NCL-MHCs, Novocastra Laboratories Ltd	-	1:450
SDHA	rabbit monoclonal antibody, D6J9M, no. 11998, Cell Signaling	1:1000	1:900
USMG5	rabbit polyclonal antibody, no. 17716-1-AP, proteintech	-	1:500
VIM	rabbit monoclonal antibody, D21H3, no. 5741, Cell Signaling	1:1333	1:500

Target	Secondary antibodies for Western Blot analysis	Dilution Western Blot	Dilution Immunohistochemistry
CYCS	horseradish peroxidase-conjugated polyclonal goat anti-rabbit antibody, no. 7074, Cell Signaling	1:2000	-
FIS1	horseradish peroxidase-conjugated polyclonal goat anti-rabbit antibody, no. 7074, Cell Signaling	1:2000	-
GAPDH	horseradish peroxidase-conjugated polyclonal goat anti-rabbit antibody, no. 7074, Cell Signaling	1:2000	-
SDHA	horseradish peroxidase-conjugated polyclonal goat anti-rabbit antibody, no. 7074, Cell Signaling	1:2000	-
VIM	horseradish peroxidase-conjugated polyclonal goat anti-rabbit antibody, no. 7074, Cell Signaling	1:2000	-

Target	Secondary antibodies for immunohistochemistry	Dilution Western Blot	Dilution Immunohistochemistry
CAV1	HRP-goat-anti-rabbit IgG, no. P0448, DAKO	-	1:150
FIS1	biotinylated goat-anti-rabbit IgG , no. BA-1000, Vector Laboratories	-	1:200
Myosin Heavy Chain fast	biotinylated goat-anti-mouse IgG, no. 115-065-146, Jackson ImmunoResearch	-	1:250
Myosin Heavy Chain slow	biotinylated goat-anti-mouse IgG, no. 115-065-146, Jackson ImmunoResearch	-	1:250
SDHA	biotinylated goat-anti-rabbit IgG , no. BA-1000, Vector Laboratories	-	1:200
USMG5	biotinylated goat-anti-rabbit IgG , no. BA-1000, Vector Laboratories	-	1:200
VIM	biotinylated goat-anti-rabbit IgG , no. BA-1000, Vector Laboratories	-	1:200
